# Effects of a High-Molecular-Weight Polysaccharides Isolated from Korean Persimmon on the Antioxidant, Anti-Inflammatory, and Antiwrinkle Activity

**DOI:** 10.3390/molecules26061600

**Published:** 2021-03-13

**Authors:** Ki Cheol Hwang, Hyun Young Shin, Woo Jung Kim, Mi Suk Seo, Hoon Kim

**Affiliations:** 1 Rafarophe Co, Venture Research Center Suite 605, 194-41, Osongsaengmyeong 1-ro, Osong-eup, Heungdeok-gu, Cheongju 28160, Korea; kicheol74@gmail.com; 2 Department of Integrated Biomedical and Life Science, Korea University, Seoul 02841, Korea; shydud0629@korea.ac.kr; 3 Biocenter, Gyeonggido Business and Science Accelerator, Gwanggyo-ro 147, Yeongtong-gu, Suwon 16229, Korea; wj0504@gbsa.or.kr (W.J.K.); sms@gbsa.or.kr (M.S.S.); 4 Skin-Biotechnology Center, Kyung Hee University, Gwanggyo-ro 147, Yeongtong-gu, Suwon 16229, Korea

**Keywords:** *Diospyros kaki*, persimmon fruit, cosmeceutical, skin function, polysaccharide

## Abstract

Persimmon (*Diospyros kaki*), a familiar and widespread fruit worldwide, is known to exhibit several physiological effects because of the presence of pharmacologically active compounds called phytochemicals. However, its high-molecular-weight compounds, particularly polysaccharides, have not been extensively studied. In this study, *D. kaki* extract (DK) was fractionated into low- and high-molecular-weight fractions (DK-L and DK-H, respectively) through ethanol fractionation, and their effects on antioxidant, anti-inflammatory, and antiwrinkle activities were investigated by an in vitro system. DK-H contained significantly higher contents of neutral sugar, uronic acid, and polyphenols compared to DK and DK-L. Furthermore, DK-H exhibited significantly improved pharmacological activities, such as antioxidant, anti-inflammatory, and antiwrinkle properties, compared to those of DK and DK-L, demonstrating that DK-H may play an important role in mediating the beneficial effects of persimmon. Sugar composition analysis and molecular characterization indicated that DK-H consisted of a galacturonic acid (GalA)-rich polysaccharide with a molecular weight of >345 kDa that mainly comprised GalA and small amounts of neutral sugar and polyphenol residues. These results suggest that the bioactive fraction DK-H is likely to be a GalA-rich pectic polysaccharide containing a small number of polyphenol residues, which may be a novel candidate in the pharmaceutical and cosmeceutical industries.

## 1. Introduction

Synthetic substances are widely used as major ingredients in cosmetic products; however, recently, a number of natural compounds have been applied as principal active ingredients in cosmetology [1]. Recent skincare interests have focused on the identification and application of novel natural sources and their extracts in cosmetics and cosmeceuticals [2]. Since ancient times, cosmetics have been used for maintaining skin hygiene and health; however, the recent concept of cosmetics usage includes not only maintaining healthy skin but also treating several skin-related disorders and preventing skin aging [3]. A number of medicinal plants are considered to have beneficial effects on skin health because of their pharmaceutically active components, such as polyphenols, flavonoids, terpenoids, lignin, tannins, and alkaloids, also known as phytochemicals [4]. However, in contrast to these low-molecular-weight compounds, the effects of high-molecular-weight compounds, particularly polysaccharides, isolated from natural sources on skin health have not been extensively investigated. In recent times, because of their beneficial effects on the skin, polysaccharides are increasingly used in the preparation of cosmetic formulations [5].

Persimmon (*Diospyros kaki*), belonging to the Ebenaceae family, is a familiar and widespread fruit, cultivated around the world. Its pulp is enriched with bioactive polyphenols including ferulic acid, *p*-coumaric acid, and gallic acid, as well as carotenoids, such as *cis*-mutatoxanthin, antheraxanthin, zeaxanthin, neolutein, cryptoxanthins, α-carotene, β-carotene, and fatty acid esters of β-cryptoxanthin [6]. Because of these active compounds, persimmon is known to possess various physiological properties, such as antioxidant [7], antigenotoxicity [8], antiobesity [9], hypolipidemic [10], and anticholesterol [11]. However, studies investigating the pharmacological activities of high-molecular-weight compounds in persimmon, particularly polysaccharides, are limited. The polysaccharides isolated from persimmon fruits and leaves have been shown to possess various therapeutic activities, including antioxidant [12], anticancer [13], anti-coagulant [14], antiosteoclastogenesis [15], and immunomodulatory [16]. However, to the best of our knowledge, the effect of persimmon polysaccharides on skin function improvement and their application as cosmeceutical agents have not been reported to date. Therefore, in the current study, we isolated the low- and high-molecular-weight fractions from persimmon extract, performed molecular characterization, and investigated their potential for cosmetic ingredients by evaluating their antioxidant, anti-inflammatory, and antiwrinkle activities in vitro.

## 2. Results

### 2.1. General Composition and Component Sugar Analysis

To increase the physiological activity of *D. kaki* extract (DK) on the skin, its low- and high-molecular-weight fractions (DK-L and DK-H, respectively) were isolated. The general composition of the fractions, including total sugar, uronic acid, and polyphenol contents, are shown in Table 1. DK and DK-L were mainly composed of total sugars, whereas DK-H was enriched with total sugars (460.6 mg/g) and uronic acid (436.9 mg/g). However, it is known that the accuracy of the phenol–sulfuric acid method for the quantification of total sugar content can be reduced when the standard and tested sample do not have a similar qualitative composition [17]. Thus, it is thought that the total sugar determination of DK-H was interfered with due to its high amounts of uronic acid and polyphenols. Interestingly, DK-H contained significantly higher amounts of uronic acid than DK or DK-L. In addition, the polyphenol contents were significantly higher in DK-H (109.6 mg/g) than in other samples.

### 2.2. Component Sugars and Their Molecular Characterization

**Figure 1:**
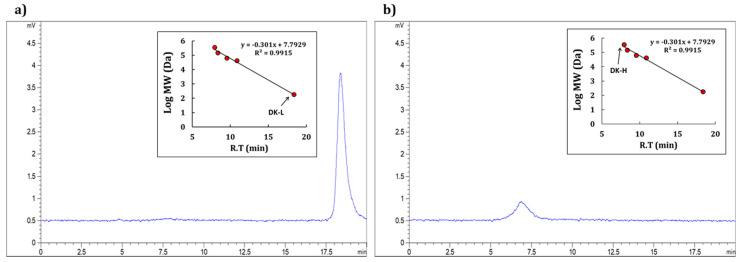
Molecular weight determination of (**a**) DK-L and (**b**) DK-H isolated from persimmon extract using high-performance gel filtration chromatography. Each fraction was loaded into the chromatographic system equipped with a Superdex 200 Increase 10/300 GL packed column and an evaporative light scattering detector (ELSD). The calibration curve was prepared with pullulan standards with different molecular weights (p-180, p-41400, p-62600, p-144000, and p-345000).

The most physiologically active sample, DK-H, was subjected to a detailed chemical composition analysis. Based on the results of the chemical composition analysis (Table 1), we analyzed the sugar composition of DK-H, its most abundant component. High-performance liquid chromatography (HPLC) analysis through the 1-phenyl-3-methyl-5-pyrazolone (PMP) derivatization of monosaccharides revealed that DK-H contains high amounts of the uronic acid, galacturonic acid (GalA, 73.2 mole%), with small amounts of neutral sugars, including arabinose (Ara), galactose (Gal), glucose (Glc), xylose (Xyl), rhamnose (Rha), and mannose (Man), with 7.4, 7.1, 5.0, 2.9, 2.3, and 2.2 mole%, respectively (Table 2). Further, the molecular weight determination analysis of DK-H indicated that it mainly contains macromolecules with an average molecular weight of >345 kDa (Figure 1a). However, unfortunately, its approximate molecular weight could not be determined exactly because it was beyond the range of the calibration curve determined by pullulans. However, DK-L mainly consisted of low-molecular-weight compounds having a molecular weight of <1 kDa (Figure 1b).

### 2.3. Antioxidant and Elastase Inhibitory Activities

The results corresponding to the antioxidant and elastase inhibitory activities of the samples are shown in Table 3. The 2,2′-azino-bis-(3-ethylbenzothiazoline-6-sulfonic acid) (ABTS) and 2,2-diphenyl-1-picrylhydrazyl (DPPH) radical scavenging activities of DK-H (IC_50_, 0.5 and 1.5 μg/mL, respectively) were found to be significantly higher than those of DK (IC_50_, 10.3 and 17.1 μg/mL, respectively) and DK-L (IC_50_, 24.0 and 32.8 μg/mL, respectively). In addition, the ferric reducing antioxidant power (FRAP) activity of DK-H (2203.8 mmol/g) was significantly higher than that of DK (65.6 mmol/g) and DK-L (19.2 mmol/g). Furthermore, the in vitro elastase inhibitory activity of DK-H was shown to be significantly higher, with an IC_50_ value of 8.1 μg/mL, than that of DK (IC_50_, 647.1 μg/mL).

### 2.4. Anti-inflammatory Activity Using RAW 264.7 Cells

**Figure 2:**
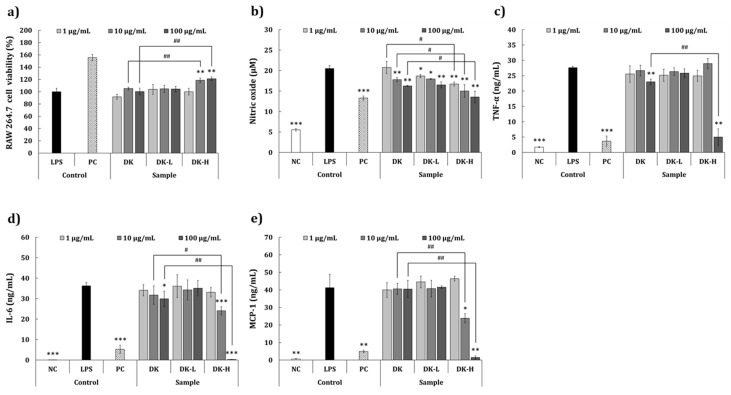
Effects of the fractions isolated from persimmon fruit on the (**a**) viability of RAW 264.7 cells and inhibition of (**b**) nitric oxide, (**c**) tumor necrosis factor-alpha, (**d**) interleukin-6, and (**e**) monocyte chemoattractant protein-1 production in LPS-induced RAW 264.7 cells. Culture medium and lipopolysaccharide (LPS; 1 μg/mL) were used as a negative control (NC) and inflammation-induced control (LPS), respectively. Treatment with dexamethasone (20 μg/mL) followed by LPS induction was used as a positive control (PC). DK, DK-L, and DK-H refer to *D. kaki* extract and its low- and high-molecular-weight fractions, respectively. Statistical differences between the groups were analyzed by Student’s *t*-test. Asterisks indicate significant differences between the sample and LPS-treated control. The crosshatch patterns indicate significant differences between DK and DK-H at the same concentrations. * and #, *p* < 0.05; ** and ##, *p* < 0.01; ***, *p* < 0.001.

The cytotoxic effect of the fractionated samples on RAW 264.7 cells was evaluated by 3-(4,5-dimethylthiazol-2-yl)-2,5-diphenyltetrazolium bromide (MTT) assay (Figure 2a). At 1, 10, or 100 μg/mL, none of the samples had any toxic effects on RAW 264.7 cells. Furthermore, DK-H significantly increased the viability of the cells at 10 or 100 μg/mL (118.5% and 121.1% against the LPS-induced control) compared to that of DK at the same doses. The inhibitory activities of the samples on proinflammatory mediators released in LPS-induced RAW 264.7 cells are shown in Figure 2b,e. As shown in Figure 2b, significant inhibition of nitric oxide (NO) production was observed in all the groups in a dose-dependent manner compared to that in the LPS-treated control group. In particular, the inhibition was significantly higher in DK-H treated cells (−25.5% to −46.6% against the LPS-treated control at 1–100 μg/mL doses) than in DK or DK-L treated cells. In addition, inhibition in NO production was significantly higher in cells treated with DK-H than those treated with DK at all doses. Furthermore, the decrease in tumor necrosis factor-alpha (TNF-α) production was not significant in cells treated with DK, DK-L, or DK-H at 1 or 10 μg/mL compared to the LPS-treated control cells (Figure 2c). However, significant inhibition of TNF-α production was observed in the 100 μg/mL DK (−17.9%)- or DK-H (−87.4%)-treated groups compared to the LPS-treated control group. In particular, the TNF-α inhibitory activity of the DK-H was significantly higher than that of the DK. The results of interleukin-6 (IL-6) inhibition are illustrated in Figure 2d. A significant inhibition in IL-6 production was observed in cells treated with 100 μg/mL DK (−17.6%); however, the most predominant inhibition of IL-6 production was observed in groups treated with 10 or 100 μg/mL DK-H (−33.7% and −99.6%, respectively) compared to the LPS-treated control group. The results of monocyte chemoattractant protein-1 (MCP-1) inhibition are shown in Figure 2e. A significant decrease in MCP-1 production was observed only in 10 or 100 μg/mL DK-H-treated groups (−48.3% and −98.0%) compared to that in the LPS-treated control group.

### 2.5. Anti-Inflammatory Activity on the Skin Using HaCaT Cells

**Figure 3:**
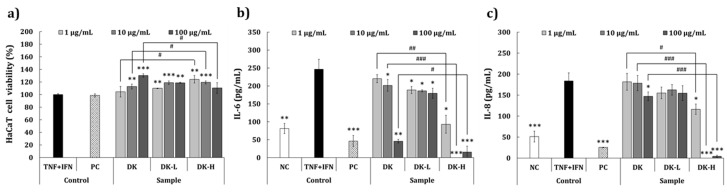
Effects of the fractions isolated from persimmon fruit on the (**a**) viability of HaCaT cells and inhibition of (**b**) interleukin-6 and (**c**) interleukin-8 in TNF-α and IFN-γ-induced HaCaT cells. Culture medium and TNF-α + IFN-γ (each 10 ng/mL) were used as a negative control (NC) and skin inflammation-induced control, respectively. Treatment with dexamethasone (20 μg/mL) followed by TNF + IFN stimulation of the cells was used as a positive control (PC). DK, DK-L, and DK-H refer to *D. kaki* extract and its low- and high-molecular-weight fractions, respectively. Statistical differences between the groups were analyzed by Student’s *t*-test. The asterisks indicate significant differences between each sample and LPS-treated control. The crosshatch patterns indicate significant differences between DK and DK-H at the same concentrations. * and #, *p* < 0.05; ** and ##, *p* < 0.01; *** and ###, *p* < 0.001.

The cytotoxic effects of the test samples on HaCaT cells treated with TNF-α and interferon-γ (IFN-γ) are shown in Figure 3a. MTT assay demonstrated that the samples had no cytotoxic effect at any of the concentrations tested. Furthermore, a significant increase in cell viability was observed in groups treated with DK (12.8% and 30.3% increase at 10 and 100 μg/mL, respectively), DK-L (10.0–19.0% increase at 1–100 μg/mL), or DK-H (24.3% and 119.4% at 1 and 10 μg/mL, respectively) compared to the TNF-α and IFN-γ (TNF + IFN)-treated control group. The inhibitory effect of the samples on IL-6 and interleukin-8 (IL-8) production is illustrated in Figure 3b,c, respectively. As shown in Figure 3b, IL-6 production was significantly inhibited by DK treatment at 10 and 100 μg/mL (−27.3% and −121.4%, respectively) but not at 1 μg/mL compared to the TNF + IFN-treated control group. IL-6 production was significantly inhibited by DK-L (−35.2% to −40.7%) and DK-H (−92.9% to −172.3%) treatments at all concentrations. Particularly, inhibition of IL-6 was better at all concentrations of DK-H compared to other samples. The inhibitory effect of the samples on IL-8 production is provided in Figure 3c. The results showed that IL-8 production was found to be significantly inhibited at all concentrations of DK-H (−50.8%, −157.8%, and −135.5% at 1, 10, and 100 μg/mL, respectively). Furthermore, a significant inhibition in IL-8 production was observed at 100 μg/mL of DK (−27.5%) compared to the TNF + IFN-treated control group.

### 2.6. Antiwrinkle Activity

**Figure 4:**
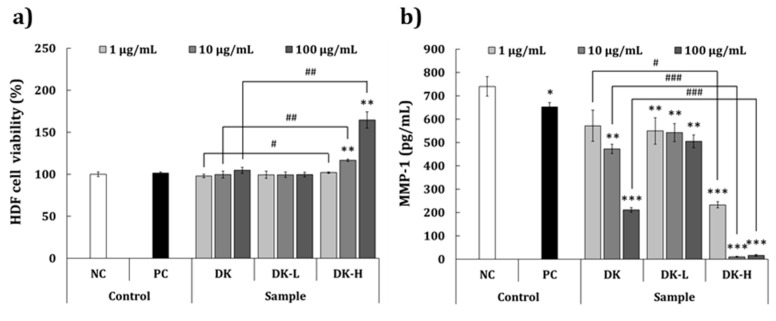
Effects of the fractions isolated from persimmon fruit on the (**a**) viability of human dermal fibroblasts (HDFa) and inhibition of (**b**) metalloproteinase-1 (MMP-1) in HDFa. Culture medium and retinoic acid (2 μg/mL) were used as a negative control (NC) and positive control (PC), respectively. DK, DK-L, and DK-H refer to *D. kaki* extract and its low- and high-molecular-weight fractions, respectively. Statistical differences between the groups were analyzed by Student’s *t*-test. The asterisks indicate significant differences between each sample and LPS-treated control. The crosshatch patterns indicate significant differences between DK and DK-H at the same concentrations. * and #, *p* < 0.05; ** and ##, *p* < 0.01; *** and ###, *p* < 0.001.

The antiwrinkle activity of the test samples was evaluated using human dermal fibroblasts (HDFa). MTT assay confirmed that the concentrations used for evaluating the antiwrinkle activity were not cytotoxic (Figure 4a). No significant difference in cell viability was observed at any of the concentrations of DK and DK-L compared to the NC group. However, DK-H at doses of 10 and 100 μg/mL significantly increased the cell viability (+16.7% and +64.7%, respectively) compared to the NC group. The effect of the samples on matrix metalloproteinase-1 (MMP-1) production is shown in Figure 4b. DK treatment significantly decreased MMP-1 level at 10 and 100 μg/mL concentrations (−36.1% and −71.5%, respectively) but not at 1 μg/mL when compared to the NC group. Furthermore, significant inhibition in MMP-1 production was observed at all concentrations of DK-L (−25.8% to −31.8%) and DK-H (−68.6% to −98.6%). The results demonstrated that the inhibitory effect of DK-H on MMP-1 expression was better than the other samples.

## 3. Discussion

Plant extracts generally contain various pharmacologically and biologically active constituents, which can be divided into two types of compounds according to their molecular weights. First, small molecules, known as phytochemicals, which include phenols, flavonoids, terpenes, and carotenoids [18]. Second, macromolecules, high-molecular-weight compounds, which are polymers, composed of multiple monomer units, including carbohydrates (especially polysaccharides) and proteins [19]. In particular, plant polysaccharides have gained great attention in the cosmetic industry owing to their several pharmaceutical effects on human skin [20,21]. Nevertheless, additional studies are required to establish a number of plant-based polysaccharides as functional cosmetic and cosmeceutical ingredients.

Persimmon, a popular and familiar fruit worldwide, possesses various pharmaceutically active compounds, such as organic acids, polyphenols, flavonoids, terpenoids, and polysaccharides [9]. However, the effects of persimmon polysaccharides on skin function improvement have not been investigated to date. Therefore, the current study aimed at evaluating the physiological properties, such as antioxidant, anti-inflammatory, and antiwrinkle properties, of water-soluble polysaccharides isolated from persimmon extract. The ethanol precipitation method has been widely used for the separation of macromolecules based on their molecular size [22]. Hence, in the present study, we isolated low- and high-molecular-weight fractions (DK-L and DK-H, respectively) from persimmon fruit extract (DK). The chemical component and monosaccharide composition analyses indicate that DK-H is mainly composed of GalA-rich polysaccharides (approximately 90 *w*/*w*%; GalA, Ara, Gal, Glc, Xyl, Rha, and Man at a molecular ratio of 14.7:1.5:1.4:1.0:0.6:0.5:0.4, respectively) with small amounts of polyphenols (approximately 10 *w*/*w*%). The GalA-rich pectic polysaccharide was previously reported from persimmon leaf [23,24] and peel [25], whereas gallic acid and catechin were reported as the most predominant polyphenol in various cultivars of persimmon fruit [26] Further, only a single broad peak observed in molecular weight determination suggests that DK-H may comprise simple macromolecules of GalA-rich polysaccharide interacting with polyphenolic compounds. Generally, plant-derived GalA-rich polysaccharides mostly correspond to pectin, a complex polysaccharide comprising a repeating linear polymer of 1,4-linked α-GalA residues (galacturonan) containing various neutral sugar residues as substituents, such as Rha, Ara, Xyl, Gal, and Glc [27]. The structural classes of pectin include homogalacturonan (HG), xylogalacturonan (XG), rhamnogalacturonan I (RGI), and rhamnogalacturonan II (RGII), and the conventional structure of pectin is portrayed as a composite structure, consisting of RGI, RGII, and XG attached within the HG backbone [27]. To identify the fine structural characteristic of DK-H, further fractionation by column chromatography, such as ion exchange and size exclusion, in addition to chemical and structural analyses, such as GC-MS, IR, and NMR, is needed in future studies [28]. Thermodynamic and spectral measurements, polymer particle tracking analysis, and microstructure observation are also required to identify the structural interaction between the GalA-rich polysaccharide and polyphenols in DK-H [29]. In addition, individual polyphenol composition in DK-H might be fully identified in the near future.

When the antioxidant activities of the fractions were compared, DK-H treatment resulted in 20.6-fold and 11.4-fold lower IC_50_ values in ABTS and DPPH free radical scavenging activity assays and a 33.6-fold higher value in the FRAP activity assay than those of the DK treatment. These results demonstrated that antioxidant activity is drastically increased when DK is fractionated into DK-H, suggesting that DK-H may be utilized as a novel ingredient for synthesizing antioxidant agents from persimmon fruit. Although a number of studies have reported the antioxidant activities of various constituents derived from different parts of persimmon plants, including fruit, leaf, and seed, to the best of our knowledge, this is the first study to evaluate the antioxidant activity of GalA-rich polysaccharides isolated from persimmon. Many plant-derived polysaccharides exert potent radical scavenging and antioxidant activities, but their underlying mechanism is not yet fully elucidated. Recently, however, based on numerous studies on the relationship between antioxidant and polysaccharide structure, three factors, such as molecular weight, uronic acid contents, and other chemical components (polyphenol or protein) in the polysaccharide fraction, are supposed to play an important role in their antioxidant activity [30].

Macrophages are responsible for the initiation, maintenance, and resolution of the inflammation process, a vital defense system against wounded tissue and foreign pathogens [31,32]. In infected or damaged tissues, macrophages initiate an inflammatory response by secreting proinflammatory mediators, such as TNF-α, MCP-1, IL-6, and NO [33]; thus, the investigation and development of anti-inflammatory therapeutic candidates have been a focus for inhibiting the proinflammatory mediators. Our results demonstrated that treatment with DK-H significantly decreased the levels of proinflammatory mediators in LPS-induced RAW 264.7 cells compared to those of DK or DK-L treatment. These results suggest that the GalA-rich polysaccharides play a crucial role in the anti-inflammatory activity of persimmon. Furthermore, the effect of the fractions on the anti-inflammatory activity of the skin was evaluated by determining IL-6 and IL-8 levels using HaCaT keratinocytes, stimulated with proinflammatory cytokines, TNF-α and IFN-γ, which are known to be associated with various inflammatory diseases of the skin [34]. We observed that the production of both IL-6 and IL-8 was significantly inhibited by DK-H treatment at all tested concentrations compared to those of DK and DK-L, demonstrating a decrease in the skin inflammation of DK when it is fractionated into DK-H. Cho et al. [35] reported the antiatopic effect of persimmon leaves and demonstrated the inhibition of ultraviolet B-stimulated chemotactic proteins (CCL2 and CCL27) in HaCaT cells. In addition, Kotani, et al. [36] reported that oral administration of persimmon leaf extract and its active ingredient astragalin could reduce the development of atopic dermatitis by suppressing scratching behavior, serum IgE levels, and several histological signs using an atopic dermatitis mouse model (NC/Nga). Nevertheless, to the best of our knowledge, this is the first study demonstrating the anti-inflammatory effect on the skin of GalA-rich polysaccharides isolated from persimmon.

Skin wrinkles, which are regarded as one of the signs of human aging, are generally known to be formed by a group of enzymes responsible for the degradation of extracellular matrix proteins, such as collagen and elastin in the skin tissue [37]. Collagenases and elastases are the key enzymes that are responsible for the breakdown of collagen and elastin fibers, resulting in skin wrinkles. Thus, the evaluation of anticollagenase and antielastase activities is considered appropriate for developing and studying the antiwrinkle efficacy of natural compounds [37]. Our results indicated that the elastase inhibitory activity of DK-H was significantly higher than that of DK and DK-L. In addition, DK-H significantly suppressed the production of MMP-1 (interstitial collagenase) compared to DK and DK-L in human dermal fibroblasts. These results suggested that the GalA-rich polysaccharides in persimmons may play a crucial role in the suppression of wrinkle formation by inhibiting MMP-1 and elastase activity. To increase the possibility of usage as a novel cosmetic ingredient, a comparative study between DK-H and other polysaccharides commonly used in the cosmetic industry, such as hyaluronic acid and tamarind, is needed for future study.

## 4. Materials and Methods

### 4.1. Preparation of Persimmon Extract and Its Fractionation

Fresh persimmons harvested in Gyeonbuk province (Sangju-gun, South Korea) in the year 2020 were obtained from the Sanju Aram Dried Persimmon Farming Association (Gyeongbuk, South Korea). The fruits were then dried for 50–60 days at room temperature. The dried persimmons (3 kg) were washed with water to remove surface impurities and then soaked in 36.64 L of purified water, supplemented with 3.36 kg of 1,3-buthylene glycol (JB Chem, Seoul, South Korea). The extraction was performed in a commercial biofeedback extractor (Daehan Media Co., Ltd., Gunpo, South Korea) by refluxing at 107 °C for 4 h, and finally, the DK with a °Brix of 11.7 was obtained. The low- and high-molecular-weight fractions of DK were obtained using the ethanol fractionation method, as described previously [24], with slight modifications. Briefly, 4 volume (*v*/*v*) of 95% ethanol was added to DK and stirred overnight at 4 °C. Following centrifugation (7000× *g*, 20 min, 4 °C), the supernatant and precipitate were separated. The supernatant was evaporated at 40 °C in a rotary evaporator (Eyela, Tokyo, Japan), followed by lyophilization using a freeze-dryer (Ilshin Biobase Co., Ltd., Dongducheon, South Korea). The precipitate was diluted with a small amount of water and dialyzed using a dialysis tubing cellulose membrane (MWCO 12,000–14,000; Sigma-Aldrich, St. Louis, MO, USA) to remove low-molecular-weight compounds. The dialyzed materials were evaporated at 40 °C and dried using an air-dryer, with the same procedures of the supernatant above. The dried supernatant and precipitate were named low-molecular-weight fraction (DK-L) and high-molecular-weight fraction (DK-H), respectively.

### 4.2. General Composition and Component Sugar Analysis

The general composition, such as neutral sugar, uronic acid, and polyphenol contents, of the samples was analyzed by the phenol–sulfuric acid [38], *m*-hydroxibiphenyl [39], and Folin–Ciocalteu [40] methods using standard references of Gal, GalA, and gallic acid, respectively. The component sugars in the samples were determined by the reverse-phase-HPLC method through PMP derivatization of the monosaccharides [41] using an Agilent 1200 series HPLC system (Agilent Technologies, Palo Alto, CA, USA) equipped with a diode array detector (DAD; Agilent Technologies, Palo Alto, CA, USA). The PMP-derived monosaccharides were then separated on the stationary phase of a YMC Triart C18 column (250 × 4.6 mm, 5 μm; YMC Co., Ltd., Kyoto, Japan) using a mobile phase consisting of 0.1 M phosphate buffer (pH 6.7) and acetonitrile (83:17, *v*/*v*) at a flow rate of 1 mL/min. The standard references, such as Ara, Xyl, Rha, fucose, Man, Gal, Glc, GalA, and glucuronic acid, used for determining component sugars were obtained from Sigma-Aldrich.

### 4.3. Antioxidant and Elastase Inhibitory Activity

The antioxidant inhibitory activities of the fractionated samples were measured by the ABTS and DPPH free radical scavenging assays, as well as a FRAP assay, whereas the elastase inhibitory activity was assessed by elastase inhibitor assay, as previously reported by Kim et al. [2].

### 4.4. Determination of Molecular Weight

The molecular weights of the samples were determined using an Agilent 1120 series HPLC system (Agilent Technologies, Palo Alto, CA, USA) equipped with an Agilent 1200 ELSD (Agilent Technologies, Palo Alto, CA, USA). The samples were separated on the stationary phase of a Superdex 200 Increase 10/300 GL column (300 × 10 mm, 8.6 μm; GE Healthcare Bio-Science AB, Uppsala, Sweden) using a mobile phase of pure water at a flow rate of 1.1 mL/min. The molecular weights of the samples were calculated using the calibration curve obtained by measuring various sizes of pullulans depending on their molecular weights (p-180, p-41400, p-62600, p-144000, and p-345000).

### 4.5. Cytotoxicity and Anti-Inflammatory Activity Using RAW 264.7 Murine Macrophages

The RAW 264.7 cells (Korean Cell Line Bank; KCLB Seoul, South Korea) were maintained in DMEM (Gibco, Bleiswijk, the Netherlands) containing 10% fetal bovine serum (FBS; Gibco) and 100 U penicillin/100 μg/mL streptomycin (P/S; Gibco) and subcultured every 2–3 days at 37 °C in 5% CO_2_ and 95% humidity. The cells at a density of 2 × 10^5^ cells/mL (200 μL) were seeded into a 96-well plate until 70–80% confluency was achieved. Then, the culture medium was removed, and 160 μL of serum-free DMEM containing P/S and 20 μL of the sample solution was added to the cells. After 30 min, 10 μg/mL of *Escherichia coli* lipopolysaccharide (LPS; Sigma-Aldrich, St. Louis, MO, USA) was added, followed by incubation for 24 h. To measure the cytotoxicity of the test samples, the culture medium was removed, and MTT (Life Technologies Co., Eugene, OR, USA) assay was performed, as previously described [42]. To measure the anti-inflammatory activities of the samples, proinflammatory mediators, such as NO (Thermo Fisher Scientific, Waltham, MA, USA), TNF-α (Thermo Fisher Scientific), IL-6 (BD Biosciences, Torreyana Rd., San Diego, CA, USA), and MCP-1 (BD Biosciences) levels were determined in the culture medium using the Griess assay or enzyme-linked immunosorbent assay (ELISA).

### 4.6. Anti-Inflammatory Activity on the Skin Using HaCaT Human Keratinocytes

HaCaT cells (CLS Cell Line Service, Eppelheim, Heidelberg, Germany) were cultured and maintained in the same media and conditions as that of the RAW 264.7 cells. The cells at a density of 1 × 10^5^ cells/mL (200 μL) were seeded in a 96-well plate until 70–80% confluency. The culture medium was replaced with serum-free DMEM and incubated for 24 h to allow the cells to be serum-starved. Then, fresh serum-free DMEM containing the test samples (180 μL) was added to the cells. After 1 h, 20 μL of a mixture of TNF-α and IFN-γ (TNF + IFN, each 10 ng/mL) was added to the cells, followed by incubation for 24 h. The cytotoxicity of the test samples was measured using MTT assay, and inflammation-related mediators, such as IL-6 and IL-8 (BD Biosciences), were determined in the culture medium using ELISA.

### 4.7. Antiwrinkle Activity Using Human Dermal Fibroblasts (HDF)

HDFa (American Tissue Culture Collection, Manassas, VA, USA) were cultured in fibroblast basal medium (Lonza, Verviers, Belgium) containing growth supplements (Lonza), such as 2% FBS, 0.1% recombinant human insulin, 0.1% gentamicin sulfate amphotericin B, and 0.1% recombinant human fibroblast growth factor-B. The cells at a density of 5 × 10^4^ cells/mL (200 μL) were seeded in a 96-well plate until 80–85% confluency. The culture supernatant was replaced with 200 μL of serum-free DMEM containing test samples. After 24 h incubation, the cytotoxicity of the test samples was measured using MTT assay, and ELISA was used to determine the level of the wrinkle-forming mediator, MMP-1 (R&D Systems), in the culture supernatant.

### 4.8. Statistical Analysis

Results are expressed as the mean ± standard deviation (SD) of three independent experiments. Statistical analyses were performed using PASW Statistics 18 (IBM Co., Armonk, NY, USA). The differences were evaluated by Student’s *t*-test or one-way ANOVA, followed by the post-hoc Tukey’s test. A *p*-value threshold of <0.05 was considered significant.

## 5. Conclusions

To enhance the antioxidant, anti-inflammatory, and antiwrinkle activities, persimmon extract (DK) was fractionated to DK-L and DK-H based on their molecular size. The high-molecular-weight fraction (DK-H) was confirmed to be a GalA-rich polysaccharide with a molecular weight of >345 kDa, comprising mainly an acidic sugar residue (GalA) and small amounts of neutral sugar residues including Ara, Gal, Glc, Xyl, Rha, and Man. These results suggest that DK-H is likely to be a GalA-rich pectic polysaccharide. Furthermore, we observed that the physiological activities, such as antioxidant, anti-inflammatory, and antiwrinkle properties of DK-H were higher than that of DK or DK-L. Our results demonstrated that GalA-rich polysaccharides, isolated from persimmon may play an important role in improving antioxidant, anti-inflammatory, and antiwrinkle activities in persimmon fruit, and DK-H may be a potential candidate for skincare products in the cosmeceutical industry. However, a small amount of polyphenol present in DK-H also has the possibility of playing a role in the expression of such activities, thus further study on the detailed polyphenol composition and content should be fully elucidated in our future work.

## Figures and Tables

**Table 1:** Chemical composition of *D. kaki* extract (DK) and its low- and high-molecular-weight fractions (DK-L and DK-H, respectively).

Sample	Total Sugar (mg Gal ^1^ Equivalent/g)	Uronic Acid (mg GalA ^2^ Equivalent/g)	Polyphenol (mg GA ^3^ Equivalent /g)
DK	230.3 ± 4.9 ^b^	27.7 ± 0.2 ^b^	1.9 ± 0.6 ^b^
DK-L	210.1 ± 2.7 ^c^	16.8 ± 0.3 ^b^	0.7 ± 0.2 ^b^
DK-H	460.6 ± 7.6 ^a^	436.9 ± 13.2 ^a^	109.6 ± 5.0 ^a^

^1^ Galactose, ^2^ galacturonic acid, ^3^ gallic acid. Different superscripts indicate statistically significant differences among the groups using Tukey’s test (*p* < 0.05).

**Table 2:** Sugar composition analysis of high-molecular-weight fraction (DK-H) isolated from *D. kaki* extract using high-performance gel filtration chromatography.

Sample	DK-H (Mole%)
Mannose	2.2 ± 0.8
Rhamnose	2.3 ± 0.0
Glucuronic acid	‒
Galacturonic acid	73.2 ± 2.1
Glucose	5.0 ± 0.3
Galactose	7.1 ± 0.3
Xylose	2.9 ± 0.3
Arabinose	7.4 ± 0.4
Fucose	‒

**Table 3:** Antioxidant and elastase inhibitory activities of *D. kaki* extract (DK) and its low- and high-molecular-weight fractions (DK-L and DK-H, respectively).

Sample	ABTS ^1^ (IC_50_^4^, μg/mL)	DPPH ^2^ (IC_50_, μg/mL)	FRAP ^3^ (mmol/g)	Elastase Inhibition (IC_50_, μg/mL)
DK	10.3 ± 0.2 ^b^	17.1 ± 2.7 ^b^	65.6 ± 1.6 ^b^	647.1 ± 68.6 ^a^
DK-L	24 ± 0.4 ^a^	32.8 ± 3.1 ^a^	19.2 ± 2.6 ^b^	n.d.
DK-H	0.5 ± 0.0 ^c^	1.5 ± 1.6 ^c^	2203.8 ± 70.8 ^a^	8.1 ± 1.1 ^b^

^1^ 2,2′-azino-bis (3-ethylbenzothiazoline-6-sulfonic acid), ^2^ 2,2-diphenyl-1-picrylhydrazyl, ^3^ ferric reducing antioxidant power, ^4^ half-maximal inhibitory concentration. Different superscripts indicate statistically significant differences among the groups using Tukey’s test (*p* < 0.05).

## Data Availability

No new data were created or analyzed in this study. Data sharing is not applicable to this article.

## Sample Availability

Samples of the crude extract prepared from persimmon fruit are available from the authors.
